# Modeling Associations between Chemosensation, Liking for Fats and Sweets, Dietary Behaviors and Body Mass Index in Chronic Smokers

**DOI:** 10.3390/nu11020271

**Published:** 2019-01-26

**Authors:** Brittany A. Larsen, Mark D. Litt, Tania B. Huedo-Medina, Valerie B. Duffy

**Affiliations:** 1 Department of Allied Health Sciences, University of Connecticut, Storrs, CT 06269, USA; brittany.larsen@uconn.edu (B.A.L.); tania.huedo-medina@uconn.edu (T.B.H.-M.); 2 Division of Behavioral Sciences & Community Health, University of Connecticut Health Center, Farmington, CT 06030, USA; litt@uchc.edu

**Keywords:** sweet liking, fat liking, e-cigarettes, body mass index, dietary behaviors, smell, taste, tobacco, cigarettes, chronic smoking

## Abstract

Chronic smokers have a greater risk for altered chemosensation, unhealthy dietary patterns, and excessive adiposity. In an observational study of chronic smokers, we modeled relationships between chemosensation, fat/carbohydrate liking, smoking-associated dietary behaviors, and body mass index (BMI). Also tested in the model was liking for sweet electronic cigarette juice (e-juice). Smokers (*n* = 135, 37 ± 11 years) were measured for: Taste genetics (intensity of 6-n-propylthiouracil—PROP); taste (NaCl and quinine intensities) and olfactory (odor identification) function; liking for cherry e-juice; and weight/height to calculate BMI. Smokers survey-reported their food liking and use of smoking for appetite/weight control. Structural equation models tested direct and indirect relationships between chemosensation, fat/carbohydrate liking, dietary behaviors, and BMI. In good-fitting models, taste intensity was linked to BMI variation through fat/carbohydrate liking (greater PROP intensity→greater NaCl intensity→greater food liking→higher BMI). Olfactory function tended to predict sweet e-juice liking, which, in turn, partially mediated the food liking and BMI association. The path between smoking-associated dietary behaviors and BMI was direct and independent of chemosensation or liking. These findings indicate that taste associates with BMI in chronic smokers through liking of fats/carbohydrates. Future research should determine if vaping sweet e-juice could improve diet quality and adiposity for smokers.

## 1. Introduction

Cigarette smoking and obesity increase disease susceptibility and all-cause mortality risk. Although U.S. cigarette smoking rates have declined, 37.8 million adults (15.5%) were current smokers in 2016 [1]. Conversely, U.S. rates of obesity have increased, with 39.8% of adults affected in 2015–2016 [2]. Chronic smoking with obesity is a complex interplay between unhealthy behaviors and biological factors that fuel both conditions and require unique attention for health promotion efforts [3]. The smoking-adiposity relationship, however, is not linear. Nicotine, a parasympa-thomimetic alkaloid in tobacco, produces an anorectic effect [4] and elevates metabolic rate [5], elucidating findings of lower body weight among smokers [6,7]. Yet two population-based studies showed that long-term smokers had a greater risk of overweight [8] and obesity [9], which may result from unhealthy dietary patterns [10,11,12]. A population-based survey showed that heavy smokers consumed a more pro-inflammatory diet (energy-dense, rich in saturated fats, added sugars, and refined carbohydrates) than did nonsmokers [10], fueling greater adiposity [13]. There also are parallels in brain reward circuitry in response to nicotine addiction and highly palatable fats and sweets, which support weight gain [14]. Of interest in the present study are associations between dietary behaviors and adiposity in chronic smokers, and whether these associations are influenced by chemosensory function, which may be altered by routine exposure to cigarette smoking.

There have been inconsistent associations between smoking and taste function in the literature, with most studies reporting effects on taste thresholds. Suprathreshold function, however, may have more applicability in efforts to understand links between taste and dietary behaviors. For example, a small trial showed that obese smokers reported less sweetness and creaminess from sugar/fat mixtures than did non-smokers and normal weight smokers [15]. Relative to non-smokers, chronic smokers from our laboratory-based study reported higher taste intensity from concentrated NaCl [16]. Variation in ability to taste the bitterness of phenylthiocarbamide (PTC) and propylthiouracil (PROP), phenotypes of genetic variation in taste, also has been studied in smokers. Cigarette smokers have been hypothesized as more likely to be PTC/PROP nontasters [17]. Smokers who are phenotypically nontasters show less aversion to the bitter taste of nicotine [18,19], may have greater nicotine dependence [20], and may be more likely to smoke based on sensory cues than smokers who are PTC/PROP tasters [21]. However, our laboratory-based study [16] and a crowdsourced cohort study [22] did not find greater frequencies of PROP nontasters among chronic smokers. Importantly, there is racial/ethnic variability in PTC/PROP tasting [23] and in cigarette smoking [1], convoluting the ability to study their intersection and effects on dietary behaviors. The olfactory function also may be influenced by cigarette smoking. A meta-analysis revealed significantly higher odds of olfactory dysfunction among current smokers [24]. Similarly, our laboratory-based study showed higher rates of hyposmia among chronic smokers from the nationally-representative 2012–2014 NHANES [16].

Of interest is how alterations in taste and smell function in chronic smokers might influence dietary behaviors and risk of excessive adiposity. Both senses play unique roles in dietary behaviors [25]. Taste function has been most commonly studied through the effects of PTC/PROP on taste and oral sensations [26], food preference [27], dietary patterns, and body weight [28]. Variation in taste function beyond PROP also has been linked to food preferences and ingestive behaviors, including salty taste [29]. Smell function plays a priming role in dietary behavior [25], cueing appetite and cephalic phase responses [30]. As reviewed, an individual’s dietary and weight response to smell impairment varies partially by self-awareness and response to the impairment [31]. Some studies have reported that smell impairment has been linked to differences in food preferences [32], while other studies have reported no substantial differences in food preferences [33] or dietary choices [34].

Obese smokers [3], especially women [35], have reported using cigarette smoking for weight control. Electronic cigarettes (e-cigarettes) are also marketed [36] and perceived as safer for weight/appetite control than tobacco cigarettes [37,38]. E-cigarettes allow the inhalation of vaporized vegetable glycerol- or propylene glycol-containing fluids (e-liquids/e-juices) that vary in nicotine concentrations and flavorings. Sweet e-juice flavors are the most popular [39], increasing the liking [40], reward and reinforcement values of e-cigarettes [41], and ability to enjoy vaping flavors that mimic sweets without ingesting calories [38]. These factors may have contributed to the substantial increase in e-cigarette usage, with global sales totaling $3.5 billion USD in 2015 [42] and may surpass $20 billion USD by 2025 [43].

Because of growing interest in overlaps between cigarette smoking, sensory cues, and dietary behaviors [14], as well as interest in e-cigarettes for appetite/weight control, we aimed to describe associations between taste and olfactory functions, food liking, smoking-associated dietary behaviors, e-juice flavor liking, and body mass index (BMI) in chronic smokers exposed to e-cigarettes. We hypothesized that chemosensory function would influence food liking, which, in turn, would influence food/beverage liking and smoking-associated dietary behaviors, which, in turn, would be associated with BMI. We used structural equation modeling to describe simultaneous associations between taste and smell function, food and sweet e-juice liking, smoking-associated dietary behaviors, and BMI among chronic smokers.

## 2. Materials and Methods

### 2.1. Participants

Purposive convenience sampling was used to recruit chronic smokers, ages 18 to 55 years, who resided in Hartford County, Connecticut. Potential participants answered newspaper and radio advertisements from May 2014 to December 2016. A telephone screening ensured that all initial exclusion and inclusion criteria were satisfied. The criteria for exclusion were: (1) Unstable medical or psychiatric disorders, including uncontrolled hypertension (blood pressure ≥160/100 mm Hg); (2) pregnancy; (3) awareness of hypersensitivity to nicotine or propylene glycol; (4) medical history of myocardial infarction(s) or cerebrovascular accident(s). The criterion for inclusion was the current use of a minimum of ten cigarettes daily. The study was approved by the Institutional Review Board (IRB) at the University of Connecticut Health Center. Participants provided informed and written consent and were compensated $20 upon completion of baseline assessments in the initial visit. Data described in the present paper were obtained from 135 participants (65 males) who completed the baseline visit.

### 2.2. Study Procedures and Measures

Eligible participants were invited for baseline laboratory testing, conducted between 10:00AM and 2:00PM. All subjects were requested to refrain from cigarette smoking for at least three hours prior to testing. After completion of the consenting process, all subjects underwent a physical examination. Current smoking status was confirmed by a breath carbon monoxide (CO) test using a Bedfont Micro+ TM Smokerlyzer handheld CO monitor (Bedfont Scientific Ltd, Harrietsham, Kent, UK). Potential participants were instructed to first inhale, hold their breath for 15 s, and then exhale slowly into the mouthpiece, aiming to empty lungs completely. CO levels are heightened in current smokers; cut-off values of ≤12 ppm can identify smokers who have refrained from smoking for at least 8 h [44]. In addition, because alcohol consumption has been linked to cigarette smoking behaviors, smokers were briefly assessed for patterns of heavy and binge alcohol consumption via a timeline follow-back method (TLFB) [45]. Heavy drinking was defined as the consumption of ≥8 drinks per week over the preceding three months while binge drinking was considered to be the consumption of ≥4 drinks during a single occasion for females or ≥6 drinks for males.

After providing consent and completion of a physical examination, participants completed the following procedures in a single visit to a hospital-based clinical research center:

#### 2.2.1. Taste and Smell Function

For the taste testing, participants were oriented, with a specific script, to the general Labeled Magnitude Scale (gLMS) and verbalized intensity ratings. The gLMS generalizes the LMS [46] to all sensations [47]. Shown in the vertical orientation on a 100-point scale, it ranges from “no sensation” (0) to “strongest sensation of any kind” (100) and includes intermediate labels at “barely detectable” (1.4), “weak” (6), “moderate” (17), “strong” (35), and “very strong” (53) [47]. Participants practiced rating the intensity of brightness of three remembered stimuli (well-lit room, dimly lit restaurant, brightest light ever seen) and then reported the intensity of 1000 Hz tone series presented in 12 dB steps from 50–98 dB. Then, participants rinsed their mouth with bottled water and then sampled tastants, served at room temperature and in plastic medicine cups. Participants reported the intensity of each tastant (1 mM quinine hydrochloride (QHCl; Sigma-Aldrich, St. Louis, MO, USA) and 1 M sodium chloride (NaCl; Morton Salt, Chicago, IL, USA), which were first drawn across the tongue apex with a medical cotton swab and then, in addition to 0.32 M NaCl, were sampled with the whole mouth per protocol via the National Health and Nutrition Examination Survey (NHANES) 2012–2014 guidelines [48]. Following this, 1 mM and 3.2 mM propylthiouracil (PROP; Tokyo Chemical Industry Co., Ltd., Portland, OR, USA) were sampled with the whole mouth. The 1 mM and 3.2 mM PROP intensities were averaged for use in the analyses. Because chronic smokers reported higher intensities from 1 M NaCl than did nonsmokers [16], only 0.32 M NaCl and averaged PROP intensities were tested in the analyses that follow. Using an algorithm previously reported [16], categorized PROP taster statuses (supertasters, medium tasters, non-tasters) were explored via chi-square to assess differences by demographics.

Smell function was measured using a 16-item odor identification task, with odors generated by an olfactometer (Osmic Enterprises, Inc., Cincinnati, OH, USA). These odor items included food (cherry, strawberry, lemon, onion, coffee, cinnamon, chocolate, grape, vanilla), warning (gasoline, smoke, menthol); and household (soap, leather, baby powder, rose) odors. Participants were instructed to lean toward the olfactometer nozzle, sniff the generated odor, and, in a forced-choice procedure, refer to four choices (one correct option and three distractors shown as pictures and word-labels), and pick the best choice. Possible scores on the odor identification task ranged from 0 to 16 correct responses for classification of: Anosmia/severe hyposmia (0–7 correct), hyposmia (8–12 correct), and normosmia (≥13 correct).

#### 2.2.2. Liking for Saturated Fats/Carbohydrates

The intensities of liking/disliking of 40 foods and beverages and 11 non-foods (physical activities, smoking products, pleasurable/unpleasurable items) were measured on a validated liking survey [49,50]. The liking survey included a bidirectional, 100-point horizontal scale labeled with five faces and verbal descriptors, which ranged from “neither like nor dislike” (0) to “strongest disliking/liking of any kind” (±100), with survey items shown as a picture and verbal descriptor. Before starting the survey, the participants were oriented to the scale (verbally and in print) with examples that represented the intensity of disliking (running out of money, paper cut), liking (winning the lottery, succeeding) and neutral (doing a routine chore). The scores on 19 of the 40 individual foods/beverage items that contribute to excessive adiposity [51] were averaged together to comprise a reliable saturated fat/carbohydrate liking index (Cronbach’s α = 0.8): Sweets and sugary beverages (doughnuts, cookies/cake/pie, chocolate, soda/sweet drinks, coffee drinks/Frappuccino^®^/ Coolatta^®^, sports drinks); high fat foods (breakfast sausage/bacon, butter/margarine, beef steak, fried chicken, whole milk, ham/pork, mayonnaise); and carbohydrates (french fries, whole wheat bread, high-fiber bar, bagel/rolls, spaghetti/pasta, high-fiber cereal). This conceptual food group was used in the analysis.

#### 2.2.3. Liking for E-juice Flavors

The e-cigarettes (Joyetech eGo-C, Shenzhen Joyetech Co., Ltd., Shajing Town, Baoan District, ShenZhen, China) were filled with e-juices (Americanliquidscore.com) comprised of a base (50% vegetable glycerin-50% propylene glycol) and 18mg/mL of nicotine alone (flavorless) or with a flavoring (tobacco, chocolate, cherry, or menthol). Participants rinsed the mouth with bottled water then blindly vaped each e-juice for one minute, presented in random order. Subjects rated the flavor-nicotine combinations for sweetness, bitterness/sourness, irritation, and level of liking/disliking on the hedonic gLMS [47]. Of the flavors tested cherry e-juice was rated highest in sweetness [52]. In the models explored here, all e-liquid flavors were tested, but only the cherry flavor showed a significant association with the variables of interest.

#### 2.2.4. Smoking-Associated Dietary Behaviors

Five items from the 68-item Wisconsin Inventory of Smoking Dependence Motives (WISDM) [53] asked participants to report tendencies to use smoking for appetite and weight control on a seven-point Likert scale (1 = “not true of me at all” to 7 = “extremely true of me”). The index of the five summed items (range 5 to 35) had a Cronbach’s α = 0.85.

#### 2.2.5. Body Mass Index

A registered nurse obtained the weight and height from each participant at baseline visit. Body mass index (BMI) was calculated as: (weight (kg) /height (meters)^2^). BMI was classified as: Underweight (>18.5), normal weight (18.5–24.99), overweight (25.0–29.99), and obese (>30). In order to create more even distributions in BMI categories, underweight participants (*n* = 3) were included in the normal weight category.

### 2.3. Statistical Analysis

Statistical analyses were conducted using Statistical Package for the Social Sciences (SPSS) version 25.0 (IBM, Armonk, New York, NY, USA), R version 3.5.0 (*R* Foundation for Statistical Programming, Vienna, Austria), and AMOS version 25.0 (IBM, Armonk, New York, NY, USA) with statistical significance criterion set at *p* ≤ 0.05. Demographic descriptors that have been previously linked with food liking or sweet preference [54], smoking-associated dietary behaviors [55], and BMI [56], were tested for differences in variables of interest (taste and smell function, food liking, sweet e-juice liking, smoking-associated dietary behaviors, and BMI). In these analyses, race/ethnicity was treated as two groups (non-Hispanic/Latino Caucasians vs. African Americans/Hispanics/Latinos/ multi-racial), which is consistent with previous research practices [57].

Independent sample *t*-tests were used to assess differences in BMI by age (younger/older than 38 years, by median split), heavy/binge drinking (yes/no), race/ethnicity (non-Hispanic/Latino Caucasian vs. African American/Hispanic/Latino/multiracial), income (≤$40,000 vs. >$40,000 annual household income), educational attainment (≤high school education/equivalent vs. some college), and marital status (single/divorced/widowed vs. married/cohabitating). One-way analysis of covariance (ANCOVA) was used to test differences in BMI by PROP taster status (non-taster, medium taster, supertaster), controlling for demographic and dietary behaviors.

The frequency distributions of BMI, the smoking-associated dietary behaviors index, and the sweet e-juice liking variable were evaluated and square root transformed. Pearson correlation analysis was used to test relationships between chemosensory function, fat/carbohydrate liking, sweet e-juice liking, smoking-associated dietary behaviors, and BMI.

Structural equation modeling (SEM) was used to test the idea that chemosensory function would influence food liking (controlling for race/ethnicity and age), which, in turn, would influence sweet e-juice liking and smoking-associated dietary behaviors (controlling for sex), which, in turn, would be associated with BMI [29,58]. A multiple imputation procedure was performed using the MICE package in R, based on restricted maximum likelihood estimation [59], to fill in missing data (<5%).

Univariate and multivariate outliers among the model variables were identified by the standardized residual (≥2.5) and by the Mahalanobis distance criteria [60]. Sensitivity analyses were conducted on original and transformed variables to assess differences in model statistics and significance [61]. Potential confounders were also included in the theoretical model, including age, breath CO readings (ppm), income, sex, race/ethnicity, and binge/heavy alcohol consumption. Confounders that were found to be non-significant were excluded from the final models. Tested associations were not found to differ significantly between the conceptual and final models, with or without the non-significant confounding variables. Measures of global fit, including χ^2^, Tucker-Lewis Index (TLI), Comparative Fit Index (CFI), and the Root Mean Square Error of Approximation (RMSEA), were chosen a priori. The criteria for adequate model fit included a non-significant χ^2^ (*p* > 0.05), TLI > 0.87, CFI > 0.92. and RMSEA < 0.05 [60]. Non-significant paths (*p* > 0.1) were trimmed from the model and the re-specified model tested, with fit parameters evaluated before being provisionally accepted [62].

## 3. Results

The sample has been described previously [16]. In brief, most were non-Hispanic/Latino Caucasians (68.1%) and 51.9% were female; this sex distribution was similar to the general distribution of the nationwide population of females (51.3%) in 2017 [63]. By race/ethnicity, our sample had fewer non-Hispanic/Latino Caucasians (68.1% vs. 74%) and Hispanics/Latinos (7.4% vs. 8.2%) and more non-Hispanic/Latino African Americans (23% vs. 7.1%) and multi-racial participants (1.5% vs. 1.3%) than the U.S. racial/ethnic distributions [63]. By BMI, our sample had significantly heavier African American/Hispanic/Latino/multiracial participants compared to non-Hispanic/Latino Caucasians (30.9 ± 6.8 vs. 27.8 ± 6.5, respectively, *p* = 0.01). Females and males did not vary by age or BMI. Compared to the general U.S. population in 2017 the smokers in our sample were less educated (42% vs. 40% with a high school diploma/GED or less), more were unemployed or retired (39% vs. 30.5% unemployed/retired), and fewer were married/cohabitating (36% vs. 56.1%) [63].

### 3.1. Body Mass Index (BMI)

Mean BMI was 28.8 ± 6.74. More of our smokers were obese when compared with the 2017 U.S. Behavioral Risk Factor Surveillance System nationally-representative sample [63] (36% vs. 31.3%, respectively) while fewer were normal weight/underweight (31% vs. 33.8%, respectively). Table 1 shows bivariate correlations between age, chemosensory function, fat/carbohydrate liking, sweet e-juice liking, smoking-associated dietary behaviors, and BMI.

### 3.2. Taste Function

The intensities of whole mouth PROP, NaCl, and quinine have been reported previously [16]. The mean intensity for the averaged 1 mM and 3.2 mM PROP solutions was 40.6±26.5 (between “strong” and “very strong” intensity), which tended to be higher in females (*t* = 1.75, *p* = 0.08). Those with heightened taste intensity of PROP were significantly more likely to be non-Caucasian (51.4 ± 28.6 vs. 35.5 ± 23.9, *t* = 3.38, *p* = 0.001) and report lower annual household income (44.5 ± 27.1 vs. 34.6 ± 25, *t* = 2.13, *p* = 0.04), but no significant differences were observed by binge/heavy alcohol consumption, age, or BMI category. The mean 0.32 M NaCl intensity was 38.3 ± 23 (“strong” intensity), which did not differ significantly by sex, age, race, income category, binge/heavy drinking, nor BMI category.

PROP taster statuses were observed to be 21% nontasters, 58% medium tasters, and 21% supertasters. Hispanic/Latino/African American/multiracial participants were significantly more likely to be supertasters (32.6%) compared to non-Hispanic/Latino Caucasians (15.6%), while significantly fewer, respectively, were PROP nontasters (11.6% vs. 25.6%; χ^2^(2)=6.72, *p* = 0.04).

### 3.3. Olfactory Function

The mean number of correctly identified odors was 12.8 ± 1.9. As reported previously, 40.7% were classified with hyposmia or anosmia/severe hyposmia [16]. Smell function was significantly poorer in those who reported a history of binge or heavy alcohol consumption (12.5 ± 1.9 vs. 13.4 ± 2, respectively, *t* = 2.69, *p* = 0.008), consistent with previous literature [64]. Smell function did not vary significantly by age, race/ethnicity, across males and females, or by BMI category.

### 3.4. Liking for Saturated Fats/Carbohydrates

Liking for saturated fat/carbohydrate foods and beverages was variable, ranging from −39.6 to +90.9, and averaging 31.4 ± 22. Older smokers reported greater liking of these foods than did younger counterparts when assessed by categorized median age of the sample (37.3 ± 21.6 vs. 24.9 ± 20.7, *t* = 3.38, *p* = 0.001). Food liking also varied by race, with greater liking ratings reported by Hispanic/Latino/African American/multiracial participants compared to non-Hispanic/Latino whites (40.7 ± 22.6 vs. 27 ± 20.4, respectively, *t* = 3.52, *p* = 0.001). Food liking also varied by BMI category (F(2, 129) = 4.95, *p* = 0.008), with obese smokers reporting the greatest overall liking ratings (38.80 ± 3.23 SEM) than normal weight/underweight (29.0 ± 3.37) or overweight (25.21 ± 3.0) smokers. There were non-significant differences in food liking between men and women or between binge/heavy alcohol consumption versus not (*p*’s > 0.05).

### 3.5. Liking for E-juice Flavors

Cherry e-liquid liking averaged 16.8 ± 32.6 and was highest in obese smokers (27.9 ± 32.7 vs. 16.5 ± 27.9 vs. 4.6 ± 33.2 reported in obese, overweight, and normal weight/underweight participants, respectively, (F(2, 127) = 6.58, *p* = 0.002). Non-whites reported a significantly higher liking for cherry e-juice than did non-Hispanic/Latino white counterparts (24.8 ± 35.74.5 vs. 13.1 ± 30.6, respectively, *t* = 2.31, *p* = 0.02). There were no significant differences in cherry e-juice liking by income, sex, age, or binge/heavy alcohol consumption.

### 3.6. Smoking-Associated Dietary Behaviors

The mean score of the smoking-associated dietary behaviors was 15.2 ± 8.4. Consistent with existing literature, female chronic smokers reported a greater tendency to use cigarettes for appetite/weight management than did males (18.1 ± 8.9 vs. 12 ± 6.5, *t* = 4.59, *p* < 0.001). Self-reported tendencies to use smoking for appetite/weight control did not differ by race/ethnicity, age, income, or binge/heavy alcohol consumption (*p*’s > 0.05). Because associations between PROP bitter phenotype and BMI may be influenced by dietary behaviors [65], we tested this association in a one-way analysis of covariance with smoking associated dietary behaviors and sex as covariates. BMI did not vary significantly by PROP taster groups (F(2, 131) = 0.093, *p* = 0.91).

### 3.7. Structural Equation Modeling of Chemosensation, Liking, Behaviors, and BMI

**Figure 1:**
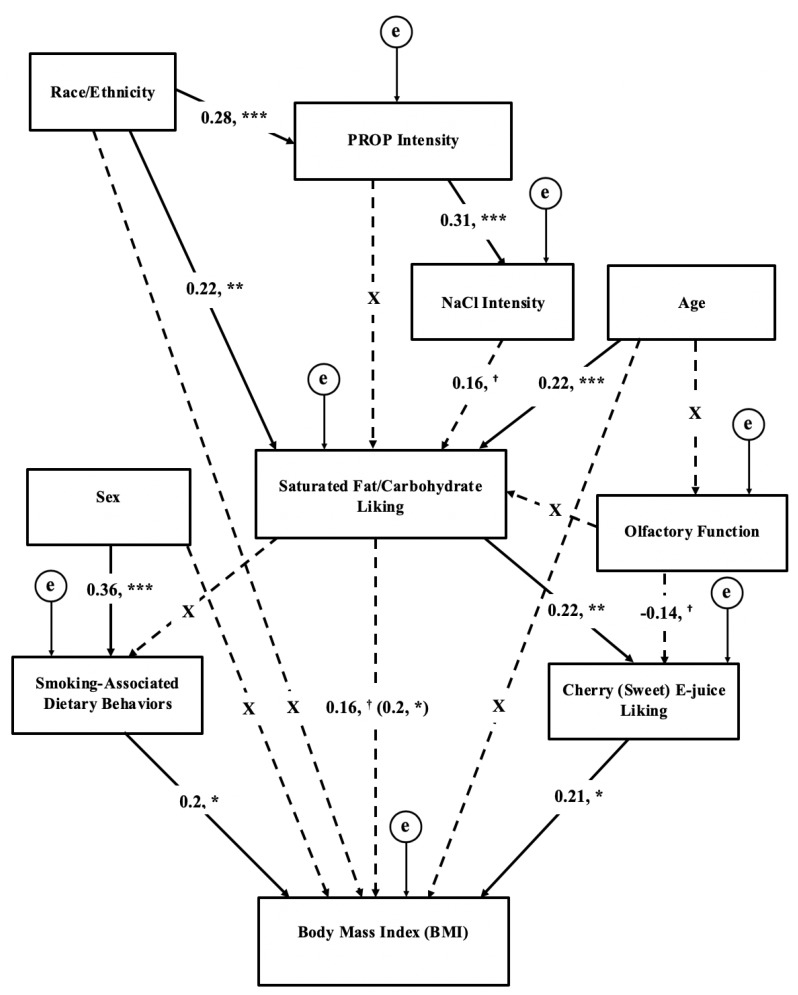
Conceptual hypothesis-based model of associations between chemosensation, liking, smoking-associated dietary behaviors, and BMI in chronic adult smokers. The numerical values labeled on the arrow lines represent standardized beta coefficients. Errors (represented by the encircled letter “e“) are required computationally, but are not of theoretical interest. The coefficient in parenthesis represents the associations before cherry e-juice liking was added to the model. indicating that cherry e-juice mediated the dietary preference-BMI relationship (indirect effect coefficient = 0.05, *p* < 0.05). Dashed lines with “X” coefficients indicate non-significant associations. The model was adequately fit (CFI = 1.00, TLI = 1.03, Chi-square = 25.6, df = 27, *p* = 0.54, RMSEA = 0.00, 90% C.I. 0.000–0.063). *** indicates that *p* ≤ 0.005; ** *p* ≤ 0.01; *p ≤ 0.05; ^†^ *p* ≤ 0.1. (PROP= 6-n-propylthiouracil).

The SEM simultaneously tested the direct and indirect associations between chemosensation, liking for saturated fats/carbohydrates, liking for sweet e-juice, smoking-associated dietary behaviors, and BMI of the hypothesized conceptual model (Figure 1). The model had excellent global fit parameters (χ^2^ = 25.6, df = 27, *p* = 0.54; CFI = 1.00; TLI = 1.03; RMSEA = 0.000, 90% C.I. 0.000–0.063), but was over-fit (TLI >1). In the model, PROP intensity and olfactory function were not directly associated with fat/carbohydrate liking (*p*’s > 0.1). Instead, NaCl taste intensity was associated with food liking (β = 0.16, *p* = 0.053), which, in turn, tended to associate with BMI (β = 0.16, *p* = 0.069). Additionally, fat/carbohydrate liking did not significantly predict smoking-associated dietary behaviors (*p*’s > 0.1). Olfactory function demonstrated tended to inversely associate with sweet e-juice liking (β = −0.14, *p* = 0.093). Finally, sex, race/ethnicity, and age did not associate significantly with BMI (*p*’s > 0.1) and were removed as covariates from the conceptual model.

**Figure 2:**
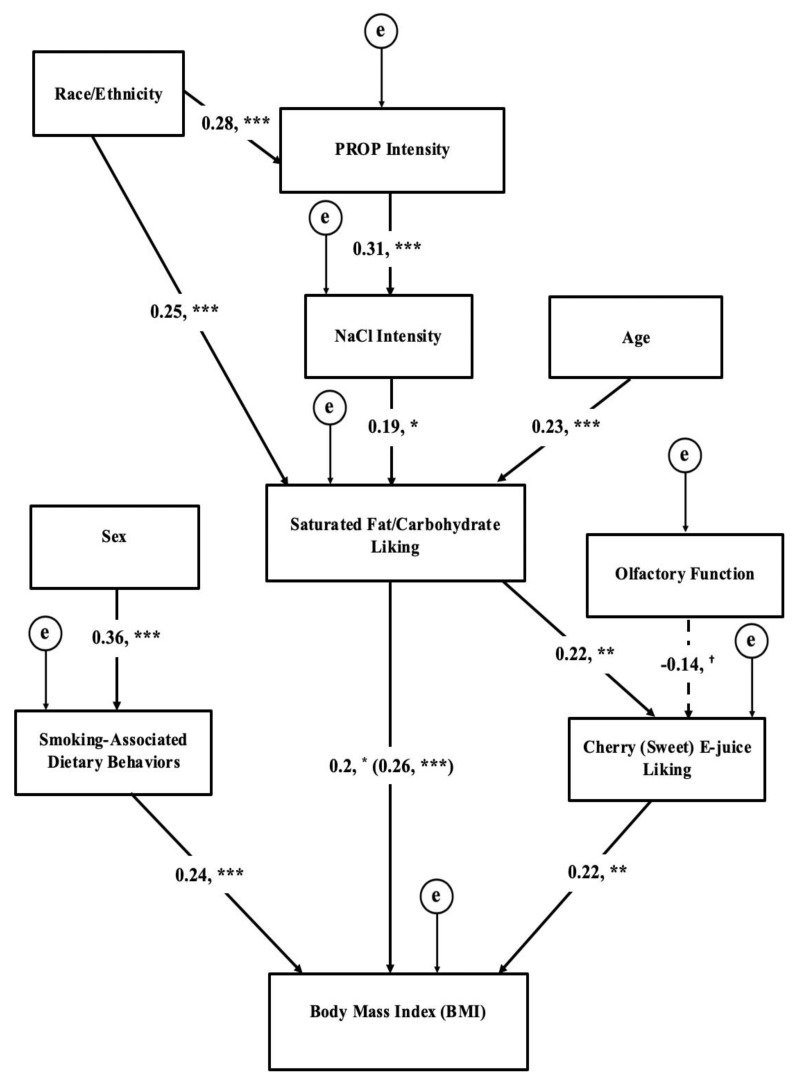
Structural equation model testing direct and indirect associations between taste, liking, smoking-associated dietary behaviors, and BMI in chronic adult smokers. Errors (represented by the encircled letter “e”) are required computationally, but are not of theoretical interest. The coefficient in parenthesis represents the associations before cherry e-juice liking was added to the model indicating cherry e-juice partially mediated the dietary preference-BMI relationship (indirect effect coefficient = 0.05, *p* < 0.05). With all non-significant pathways removed, the model remained an adequate fit (CFI = 1.00, TLI = 1.00, Chi-square = 34, df = 34, *p* = 0.47, RMSEA = 0.00, 90% C.I. 0.000–0.063). *** indicates that *p* ≤ 0.005; ** *p* ≤ 0.01; * *p* ≤ 0.05; ^†^ *p* ≤ 0.1. (PROP = 6-n-propylthiouracil).

Based on findings from the tested conceptual model and supporting bivariate analyses, a re-specified model with all non-significant pathways (*p* > 0.1) trimmed was tested in SEM (Figure 2). The global fit remained excellent and showed an improvement in the TLI, which showed a good fitting model (χ^2^ = 34, df = 34, *p* = 0.47; CFI = 1.00; TLI = 1.00; RMSEA = 0.000, 90% C.I. 0.000–0.063). In the final model, PROP intensity was related to 0.32 NaCl taste intensity, which was linked to food liking. Furthermore, food liking predicted liking for sweet e-juice and BMI, but not smoking-associated dietary behaviors. In the final model, olfactory function tended to inversely associate with sweet e-juice liking (β=−0.14, *p* = 0.089), which, in turn, partially mediated the association between food liking and BMI. Smoking-associated dietary behaviors were also found to predict BMI, but separately from chemosensory or liking variables.

## 4. Discussion

In this observational study we sought to model associations between chemosensory function, food and beverage liking, smoking-associated dietary behaviors, and body mass index (BMI) among a sample of chronic smokers. Furthermore, we modeled the interplay between liking for a vaped sweet e-cigarette juice flavor, liking for foods and beverages, and BMI. The best fitting model had measures of taste intensity that were associated with variability in fat and carbohydrate liking, which, in turn, was associated with variability in BMI. Ability to taste PROP bitterness did not show a direct association with fat and carbohydrate liking, and instead was associated through the intensity of NaCl. Greater fat/carbohydrate liking was associated with greater BMI, an association partially mediated by greater liking of the vaped sweet e-juice, suggesting that liking for sweetness, even in an e-juice, was a primary determinant of BMI in this sample. Olfactory function, measured by an odor identification task, failed to associate significantly with the liking variables. Finally, reported use of smoking to control appetite and weight showed a separate pathway of association from either chemosensory or liking variables.

The sample of chronic smokers showed sufficient variability to test these models of association, including demographic, chemosensory, food liking, dietary behavior, and BMI characteristics. The study sample was gender-balanced and diverse in race/ethnicity, education, household income, and employment consistent with characteristics of smokers in the U.S. [1]; these demographic variables have associated with chemosensory function [66] and dietary behaviors [67,68]. In addition, our sample of chronic smokers captured a range of BMI from underweight to obese, with a higher frequency of obesity than that reported for adults in the U.S. [63], but consistent with greater odds of obesity in chronic smokers [9].

The observed variation in perceived PROP bitterness across the sample was similar to what we have seen previously (e.g. [69]) and that reported by other laboratory-based studies (e.g. [70]). The frequency of PROP nontasters, medium, and supertasters in our sample [16] was comparable to theoretical rates (i.e., 25% nontasters, 50% medium tasters, 25% supertasters). Greater PROP bitterness is linked with heightened ability to perceive oral sensations and tastes from fat, which may be explained in part by a higher density of fungiform papillae found on the tongue of PROP tasters compared to nontasters [71]. Among nonsmokers, this has furthermore been associated with a lower liking for fats/sweets among PROP tasters compared to nontasters [72,73,74]. Although we observed a positive bivariate association between PROP bitterness and fat/carbohydrate liking, as previously observed in young adults with poor dietary behaviors [75], perceived PROP bitterness did not contribute directly to fat/carbohydrate liking in the full structural equation model.

Instead, we observed that PROP taste intensity was indirectly associated with the liking of fats/carbohydrates through the intensity of NaCl. The PROP-NaCl association is consistent with the previous reports [29], but the ability of 0.32 M NaCl to associate with food liking among chronic smokers is a new finding. Chronic smoking likely diminishes the ability of PROP to serve as a marker for differences in oral sensation to associate with dietary behaviors and health outcomes [26]. Most studies of taste phenotype, diet, and health are conducted with non-smokers and study groups who are homogenous in race/ethnicity. As expected among chronic smokers, our study sample was diverse in race/ethnicity, which also could have contributed to a lack of direct association between PROP bitterness perception and food liking. We found more PROP supertasters among the Hispanic/Latino/African American/multiracial smokers and more nontasters among non-Hispanic/ Latino Caucasians. This is consistent with the global distribution of *TAS2R38* receptors gene mediating the ability to taste PROP and PTC bitterness [76]. Thus, there likely was a race/ethnic interaction between PROP tasting and food liking as reported previously [77].

We also tested the ability of smell function to influence liking variables and BMI. In the bivariate association, greater functioning associated with a lower liking of fat/carbohydrate foods and beverages, as well as a lower liking of sweet e-juice. However, in the final model, we could not detect variability in reported food liking based on performance on an odor identification task. However, our sample showed an overrepresentation of hyposmia and lower frequency of less severe dysfunction (severe hyposmia, anosmia) compared to the distributions reported in the nationally-representative U.S. NHANES sample [66]. This may have resulted in insufficient variability in olfactory function to associate with differences in food liking and dietary behaviors. In addition, although chronic smoking increases the risk of olfactory dysfunction [24], our study sample was younger than the age associated with declines in olfactory function [66].

The smokers in the current study varied in liking for less healthy foods (fat/carbohydrates) and greater liking was associated with greater BMI, which remained significant after controlling for age and race/ethnicity. These findings suggest that not all chronic smokers have unhealthy eating behaviors. A positive association between a liking for fat/carbohydrate foods and BMI is consistent with findings from our laboratory [78,79,80] and others [81,82]. This relationship may be pronounced in heavy smokers; nicotine, as the predominant addictive constituent and reinforcing property in tobacco products, is the primary driver of repeated cigarette smoking secondary to the neurological rewarding effects [83], especially with chronic nicotine exposure [84].

In our model, we found that some of the association between food liking and BMI was partially mediated by liking for the sweetest (cherry) nicotine-containing e-juice flavor. As expected, food liking was found to influence the liking for the sweet e-juice flavor, which, in turn, associated with BMI. The physiological effects of nicotine on appetite and weight [4,5], delivered through e-cigarettes (which are considered safer than tobacco cigarettes [85]), could provide a tool for chronic smokers with greater BMI to achieve and maintain a healthy weight. Although e-juice flavors are perceived retronasally [86], one with a greater preference for sweets may be more drawn to e-juice flavors that simulate sweet flavors [87], which may explain why the other vaped nicotine-containing e-juice flavors (chocolate, unflavored, menthol, tobacco) was unable to mediate the relationship between food liking and BMI. Such an association was only found in cherry, which was reported as the sweetest e-juice flavor choice amongst participants. Sweet flavors appear to elicit a stronger response in the nucleus accumbens (the predominant reward center) than non-sweet flavors [88]. With nicotine-containing e-cigarette sweet flavors, a supra-addictive response in the reward center of the brain was observed via fMRI. Thus, sweet e-juice flavors may be the predominant driver of the reinforcing effects of nicotine in e-cigarettes as a result of heightened neurological responses in the reward center [88]. There also may be a genetic susceptibility to the reward of sweets among smokers [89]. Furthermore, artificial sweeteners are commonly included in the ingredients of e-juice flavors [87]. Consumption of these sweeteners is high in the U.S., especially among individuals with obesity [90]. A greater level of scientific evidence is needed [91] to address the active debate on whether or not these sweeteners support weight management [92] or fuel the risk of obesity and associated chronic disease [93]. Finally, our model also demonstrated an inverse tendency between olfactory function and sweet e-juice preference. With the insufficient representation of olfactory impairment among our smokers, we were unable to fully test the olfactory function, sweet liking, and sweet e-juice relationships.

Smoking-associated dietary behaviors were associated with BMI in our statistical model without interacting with any of the liking variables. These findings are consistent with prior reports that suggest a separation of pathways between liking and wanting in the brain and that these circumstances for a stimulus, consequently, do not co-depend [94]. Of note, variability of self-reported smoking-associated dietary behaviors in our sample showed variability similarly to what has been previously reported [95]. The greater obesity and overweight amongst our sample may explain the elevated levels of using smoking to control appetite and weight in our sample, as overweight and obese individuals are more likely to use smoking for appetite/weight control [3]. Our findings were consistent with differences observed in smoking-associated dietary behaviors between males and females, with females reporting higher mean tendencies to smoke for appetite and weight control [95].

This current study is not without limitations. The observational cross-sectional nature of this study cannot be used to draw cause-and-effect relationships. In addition, the range of e-cigarette flavors tested was rather narrow. Furthermore, because our study sought to assess associations between chemosensation, diet behavior and BMI in smokers, we did not compare our findings to non-smokers, which limits the generalization of our findings. An additional limitation is that we did not attempt to measure dietary intake directly (e.g., food frequency questionnaire, biomarker). However, measuring usual dietary behaviors by asking what is liked/disliked is a novel and feasible alternative to intake reporting which is often biased [96]. Reported food liking correlates with reported intake [69,97] and biomarkers of dietary intake and/or adiposity young adults [69,78], and adults [50,79]. Liking survey responses can be formed into an index of diet quality (similar to the Healthy Eating Index) that explains the variability in adiposity or cardiovascular disease risk factors [49,69,78]. Additionally, body composition was not analyzed beyond the calculation of BMI, which may have resulted in an overestimation of obese and overweight classifications among the sample [98]. However, chronic smokers have been reported to have a less healthy lifestyle than nonsmokers and lighter smokers [12]. Because BMI has been found to correlate highly among more sedentary individuals [98], however, the risk for overestimating overweight and obesity in our sample is not likely to be significant. Finally, a laboratory procedure may not faithfully reflect true preferences and behaviors with respect to vaping, eating, and BMI.

## 5. Conclusions

This observational study supports the idea that variation in taste perception associates with variation in fat/carbohydrate liking. Food liking, in turn, was associated with some of the variations in BMI amongst chronic smokers. Moreover, liking for sweet e-juice flavors partially mediated the association between food liking and BMI. The associations between taste, food liking, and BMI were separate from the associations between reported use of smoking to control appetite/weight and BMI. Dual chronic smoking with obesity presents a greater risk of further chronic conditions and diseases than either health risk alone. Chronic smokers can lose weight comparable to nonsmokers in a weight loss intervention [99]. The present study provides observational findings that sweet e-cigarettes may attenuate some of the association between greater liking of sweets and high-fat foods/beverages and greater BMI. Prospective studies are needed to test whether chronic smokers with obesity would benefit from the availability of sweet e-cigarette e-juice flavors in order to satisfy their liking for less healthy foods and assist in weight control.

## Figures and Tables

**Table 1:** Bivariate correlations among variables used in structural equation models in chronic smokers (*n* = 135) ^1^.

Variable Number	Variable	1	2	3	4	5	6	7	8
**1**	**Age**	1							
**2**	**PROP intensity**	0.05	1						
**3**	**0.32 NaCl intensity**	0.09	**0.32 ^c^**	1					
**4**	**Fat/carb liking**	**0.29 ^c^**	**0.18 ^a^**	**0.22 ^a^**	1				
**5**	**SDBI**	0.11	0.08	0.01	−0.11	1			
**6**	**Olfaction**	−0.12	0.06	−0.06	**−0.18 ^a^**	0.03	1		
**7**	**Sweet E-J liking**	0.12	−0.01	0.03	**0.26 ^c^**	−0.02	**−0.19 ^a^**	1	
**8**	**BMI**	0.17	0.07	−0.07	**0.24 ^b^**	**0.22 ^a^**	−0.02	**0.27 ^c^**	1

^1^ The bolded correlation coefficients were statistically significant, where PROP intensity = perceived taste intensity of 6-n-propylthiouracil, a probe for genetic variation in taste, SDBI = Smoking Dietary Behavior Index [53], Sweet E-J Liking = Sweet E-juice Liking, and BMI = Body Mass Index. ^a^ correlations were significant at *p* ≤ 0.05; ^b^ *p* ≤ 0.01; and ^c^ *p* ≤ 0.005.
